# Unveiling the potential of extracellular and outer membrane vesicles in fish aquaculture: a comprehensive review

**DOI:** 10.3389/fimmu.2026.1793740

**Published:** 2026-08-24

**Authors:** So Young Park, Md Tawheed Hasan, Dong Nyoung Oh, Ga Yeong Kim, Jong Min Lee

**Affiliations:** 1 Department of Biotechnology, Pukyong National University, Busan, Republic of Korea; 2 Department of Aquaculture, Sylhet Agricultural University, Sylhet, Bangladesh

**Keywords:** aquaculture, disease diagnosis, extracellular vesicles, immune response, outer membrane vesicles

## Abstract

Live eukaryotic cells produce extracellular vesicles (EVs) that play a crucial role in cell-to-cell communication and transport various cargoes, including proteins, nucleic acids, and lipids. Based on their biogenesis, size, content, and pathways, EVs are mainly categorized into exosomes, microvesicles, and apoptotic bodies. These EVs have been studied as promising biomarkers for disease diagnosis, stress responses, and indicators of immune-physiological health in prominent aquaculture fish species. In fish studies, EVs have been associated with sex determination and immune response regulation, whereas EV-mediated tissue regeneration and cell permeability have mainly been investigated using zebrafish or medaka models, including studies employing non-fish- or cell culture-derived EVs. On the other hand, outer membrane vesicles (OMVs), primarily produced by gram-negative bacteria, possess acellular properties and are being explored for vaccine development. Intraperitoneal injections of OMVs act as vaccines, providing protection against infectious diseases and mortality. OMVs also modulate immune responses through gene transcription and promote anti-pathogenic antibody production. However, OMVs, which are biologically active nanoparticles, demonstrated infection potential in cultured species. Moreover, hemolytic activity, biofilm formation to protect the parent cell, and cytopathic effect were also confirmed in fish pathogen OMVs. Despite limited research on EVs and OMVs in fish, their potential in aquaculture should not be overlooked. In this report, we discuss the content and mechanisms of eukaryotic and prokaryotic nanovesicles in fish, along with the properties mentioned above. Moreover, research gaps and the future prospects of these nanomaterials will open a new prospect for upcoming researchers to utilize EVs and OMVs in the aquaculture sector.

## 1 Introduction

Aquaculture involves the cultivation of freshwater, brackish, and saltwater aquatic populations under controlled and semi-natural conditions. This form of farming encompasses breeding, harvesting, and rearing stages of various aquatic organisms, including fish, shellfish, algae, and mollusks. The primary purpose of aquaculture is to meet the growing global demand for animal protein. Aquaculture offers several benefits, including efficient food production, a lower environmental impact compared to land-based farming, contributions to economic growth, habitat restoration, and reduced carbon emissions (1, 2). However, the intensification of the aquaculture industry—due to factors such as overstocking, feed wastage, and inadequate water exchange can lead to environmental degradation and stress on cultured animals (3, 4). Stress negatively affects the immune-physiological status of these animals, making them susceptible to opportunistic and pathogenic microbes, which can cause infectious disease outbreaks on aquaculture farms (5–10).

Addressing stress and infectious diseases is crucial in modern aquaculture. Efforts to reduce these issues include optimizing environmental conditions (11, 12), implementing effective monitoring and feed management practices (13–15), using probiotics (16–19), multibiotics (20–23), and phage therapy (24), and adopting proper handling methods (25, 26). However, despite these measures, results have not always been satisfactory. Challenges remain, such as limitations in vaccines administration (including stress and inadequate antibody production) and the potential negative consequences of antibiotic use (such as mass mortality of beneficial microbes, antibiotic-resistant pathogens, and residue in fish that can be transmitted to humans) (27, 28). Additionally, ensuring the viability of probiotics in feed, aquatic environments, and harsh fish intestinal environment are also factors that aquaculture scientists are considering (18, 22, 29). In recent times, extracellular vesicles (EVs) and outer membrane vesicles (OMVs) have drawn the attention of the aquaculture scientific community for their potential as stress markers (30, 31), in wound healing by tissue regeneration (32), as indicators of immune response regulation (33, 34), carriers of natural and artificial products (35), sex identification (36), mortality protection (37–39), disease diagnosis (40–42), and biofilm formation (43, 44).

EVs are released by eukaryotic cells into the extracellular space, surrounded by a lipid membrane. These tiny particles play a crucial role in intercellular communication. EVs can be categorized into three main types: exosomes, microvesicles, and apoptotic bodies (45, 46). Exosomes are typically 30–150 nm, microvesicles are approximately 50–1,000 nm, and apoptotic bodies are generally 1–5 µm in diameter, although these size ranges may overlap depending on the cell source and isolation method. Their classification is based on functional activities, biogenesis, content, size, and release pathways. EVs facilitate communication between cells, participate in various physiological and pathological processes, and transport proteins, nucleic acids, lipids, and other organelles from parent cells to target cells (47).

In contrast, gram-negative bacterial outer membranes release spherical structures known as OMVs. These OMVs contain a diverse array of biomolecules, including lipids, proteins, and nucleic acids. OMVs play critical roles in bacterial survival and interactions with host cells (48) Unlike exosomes and microvesicles, OMVs have distinct biogenesis pathways and functions that have been extensively researched (49). They carry various toxins, virulence factors, and bioactive proteins, contributing to interactions between bacteria and their hosts (50). Studying OMVs is essential for understanding their roles in disease progression and their potential applications in infectious disease diagnosis and mortality control (51).

Traditionally, aquaculture research has primarily centered around nutrition, genetics, and disease management. However, recent advances in the field of EVs and OMV biology have revealed a fascinating narrative regarding the roles of these nanoscale particles in intercellular communication, immune modulation, disease control and diagnosis, and environmental adaptation within aquatic ecosystems. EVs and OMVs have been investigated in various fish and aquatic species for multiple applications, including immunization, stress monitoring, vesicle characterization, biofilm formation, vaccine development, and host immune regulation. Previously, EVs were utilized for immunization of Nile tilapia (*Oreochromis niloticus*) and Atlantic cod (*Gadus morhua*) against francisellosis (52). Additionally, EVs isolated from *G. morhua* plasma and serum were found to contain elevated levels of microRNA (miRNA)-21 and miRNA-155 under higher temperature stress (30). Zhao and colleagues (35) summarized EVs extracted from various aquatic animals, covering extraction methods, identification, characterization, and content analysis. On the other hand, OMVs spherical structures released by gram-negative bacteria—play essential roles in biofilm formation (43). Vaccination with *Francisella noatunensis* OMVs has been shown to reduce francisellosis in zebrafish (*Danio rerio*) (37), while *Vibrio ordalli* OMVs exhibit hemolytic enzyme activity (53). Ellis and Kuehn (54) proposed the virulence and immune modulatory functions of OMVs. Furthermore, small RNA (sRNA)-enriched OMVs from *Flavobacterium psychrophilum* interact with immune gene transcription in rainbow trout (*Oncorhynchus mykiss*) (55). Despite these intriguing findings, no comprehensive reviews have yet been published focusing on the potential analysis of EVs and OMVs in important fish species. Such an analysis could encompass stress reduction, tissue regeneration, host-pathogen interactions, immune enhancement, and disease prevention and diagnosis.

This review aims to explore the contents and mechanisms of EVs and OMVs, highlighting their untapped potential in fish tissue regeneration, immune response regulation, sex identification, disease diagnosis, and stress response. Additionally, we delve into the properties of OMVs related to fish mortality protection, immunity modulation, biofilm formation, and cell cytotoxicity. By synthesizing existing knowledge and identifying gaps, this review not only illuminates the current state of research but also provides a roadmap for future investigations.

## 2 Classification and contents of EVs and OMVs

Beyond their classification based on size and biogenesis, EVs also exhibit distinct functional properties depending on cellular origin, molecular cargo, release pathway, and biological context. In addition to exosomes, microvesicles, and apoptotic bodies, other EV types, such as stressed EVs, autophagic EVs, and matrix EVs, possess specific characteristics and functions (47). Proper EV classification is crucial for understanding their roles in intracellular communication, disease processes, and their potential as biomarkers and therapeutic tools (56). Apoptotic bodies, typically sized from 1 to 5 µm, are larger than exosomes and microvesicles and are generated during programmed cell death. Microvesicles and exosomes typically range from 50 to 1,000 nm and 30 to 150 nm, respectively (57). They originate through budding from multivesicular endosomes (for exosomes) and ectocytosis (for microvesicles). The molecular cargo of EVs provides an important basis for understanding EV function. EV cargo includes lipids, nucleic acids, and a diverse group of proteins, which contribute to plasma membrane function, cytosol dynamics, and lipid metabolism. These components govern intracellular communication and influence the fate of local and distant tissues (46). Among EVs, exosomes have been shown to mediate intracellular communication by transferring various biomolecules from donor to acceptor cells. Following cargo transfer, EV uptake by acceptor cells determines whether EV-associated molecules can exert biological effects. The delivery of EV cargo within acceptor cells has been demonstrated and quantified. However, EV uptake remains a low-yield process, and cytosolic release of EV content in acceptor cells requires endosomal acidification while being inhibited by specific factors (58, 59). Advanced scientific techniques are essential for separating and characterizing different EV types, allowing us to unravel the complex properties of these vesicles.

OMVs are nanoscale spherical structures released by gram-negative bacteria. They are classified based on certain bacterial groups, such as the Actinobacillus-Haemophilus-Pasteurella group (60). This classification is useful because OMV composition and biological function can vary depending on the taxonomic group, outer membrane structure, and physiological characteristics of the parent bacterium. OMVs are a subtype of EVs, but not all EVs are OMVs. OMVs are specifically categorized as a type of EV based on their biogenesis and composition. OMVs carry a cargo of proteins, bioactive toxins, lipids, and other molecules. In contrast, EVs transport proteins, nucleic acids, lipids, and even organelles from parent cells (61). The protein content of OMVs includes components from the outer membrane, periplasm, and cytoplasm. Lipids in OMVs consist of phosphatidylglycerol, phosphatidylethanolamines, and lipopolysaccharides, reflecting the composition of the parental bacterial outer membrane (62). OMVs are specific to gram-negative bacteria, whereas EVs can be released by diverse cell types. Biological molecules within OMVs, such as nucleic acids, ion metabolites, and signaling molecules, can be transferred to host cells (51, 63, 64). These molecules play roles in adherence to the host, stress response, biofilm production, antibiotic resistance, modulation of host innate and adaptive immune responses, and inter- and intraspecies delivery. Therefore, the distinction between EVs and OMVs should be understood not only in terms of cellular origin, but also in terms of vesicle biogenesis and cargo composition. The molecular structure of OMVs and their cargo contribute to diverse biological and pathophysiological functions, making them both offensive weapons and beneficial entities with potential implications for disease pathogenesis and bacterial survival (60).

## 3 EVs and OMVs mechanism of approach

**Figure 1:**
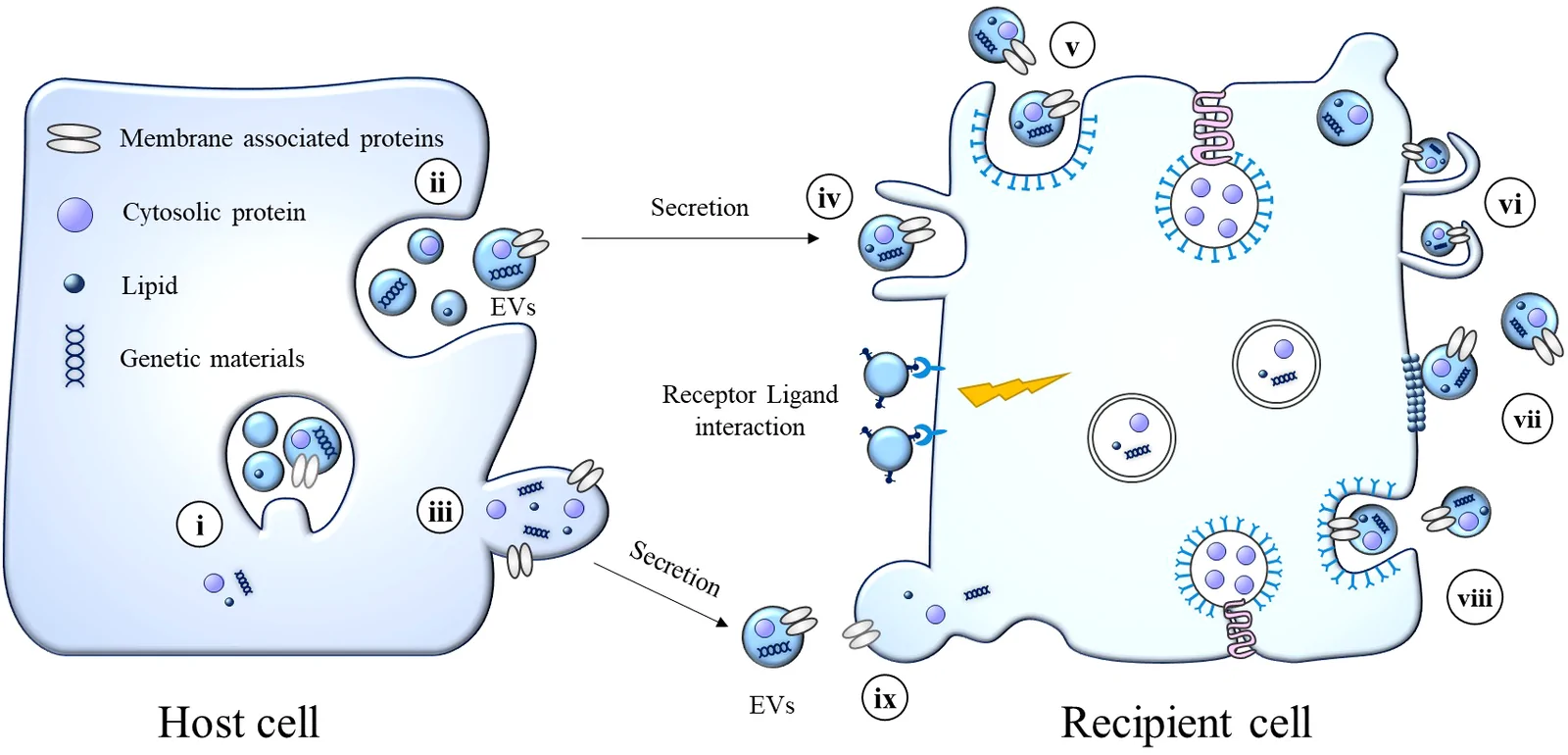
Schematic diagram of extra cellular vesicles (EVs) biogenesis from host cell i) selection of cargo materials like membrane associated protein, cytosolic protein, genetic materials, and lipid, ii) exosomes formation by inward budding of multivesicular bodies (MVB) after fusion with plasma membrane, iii) Microvesicles are produced by outward budding through fission of plasma membrane and recipient cell uptake, iv) phagocytosis, v) caveolin-mediated endocytosis, vi) micropinocytosis, vii) lipid raft-mediated endocytosis, viii) clathrin mediated endocytosis, and ix) direct membrane fusion.

EVs and OMVs transport bioactive molecules to maintain cellular communication and influence recipient cell behavior. Their physiological and pathological activities highlight their potential for therapeutic use and disease diagnosis. EVs have been effective diagnostic tools for diseases like cancer, lung disease, tissue injury, and metabolic disorders. Their application in drug delivery and therapeutic interventions in vertebrates represents a significant scientific achievement (65, 66). EV messages to recipient cells are transferred through various mechanisms, including vesicle-cell fusion, cell surface ligand signaling, and endocytosis (56, 67). EV functionality is modulated by their interaction and entry into host cells, signal transduction through interaction with cell surface receptors, and entry of EV contents into the cell cytoplasm. Common modes of EV entry into recipient cells include endocytosis and fusion with the cell plasma membrane. EV biogenesis starts with cargo material selection and accumulation, followed by inward and outward budding of multivesicular bodies (MVBs) and plasma membrane fission (68). Recipient cell EV uptake is performed through phagocytosis, caveolin- and clathrin-mediated endocytosis, micropinocytosis, and direct membrane fusion (**Figure 1**) (58, 69). EV uptake mechanisms depend on the recipient cell type, physiological conditions, and the recognition of ligands on the EV surface by cell surface receptors (70). To establish therapeutic applications, scientists have engineered EVs to insert specific therapeutic proteins that are transported to specific locations for disease treatment (71). Additionally, strategies such as grafting EVs with nanoparticles to improve kinetics and targeting and modifying nucleic acids are employed for therapeutic purposes (72, 73).

**Figure 2:**
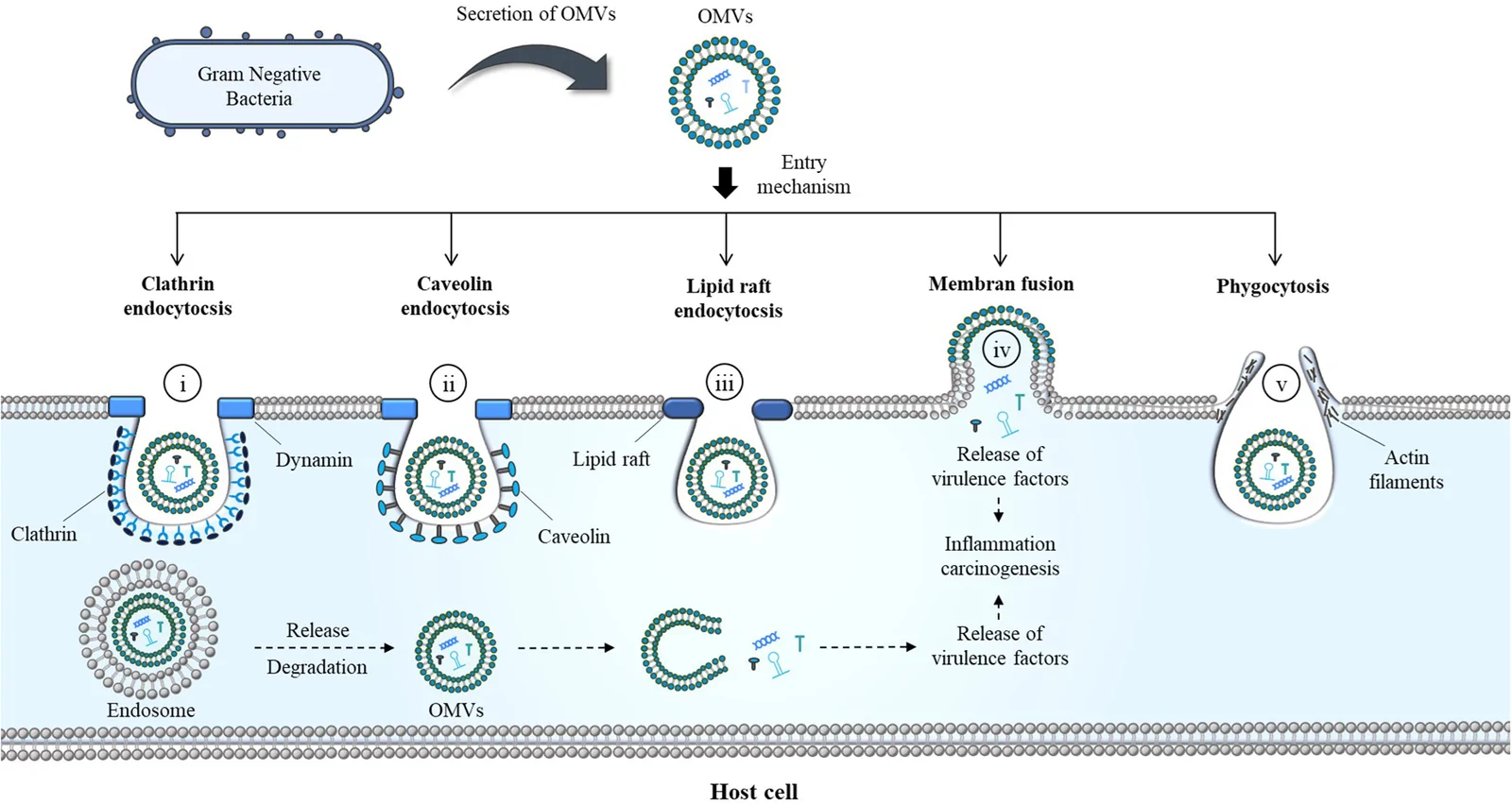
Schematic diagram of gram-negative bacteria secreted outer membrane vesicles (OMVs) entry in host cell i) clathrin mediated endocytosis, ii) caveolin-mediated endocytosis iii) lipid raft-mediated endocytosis, iv) membrane fusion, v) phagocytosis.

OMVs play various roles in bacterial physiology, including removing toxic compounds from bacteria under stress, delivering biomolecules to host cells during infection, and sequestering antibiotics and antibodies. They act as delivery vesicles, contribute to bacterial activity and survival, help create microbial communities, and maintain virulence factors. OMVs interact with environmental conditions, accumulate nutrients, facilitate gene transfer, participate in quorum sensing, and disseminate bacterial products (48, 61). Similar to EVs, OMVs enter host cells via mechanisms such as receptor-mediated endocytosis and fusion. Research suggests that OMV entry into host cells is typically governed by clathrin-mediated endocytosis and the utilization of toxin receptors, enhancing cargo delivery (50). Other pathways identified for OMV entry include lipid raft- and caveolin-mediated endocytosis (74), membrane fusion, and phagocytosis (75), with OMV composition influencing entry mechanisms (**Figure 2**). Additionally, the interaction between OMVs and host cells is partially temperature-dependent, with lower interaction activity observed at 4 °C compared to 37 °C (76).

## 4 EVs application potential in fish

### 4.1 EVs involvement in fish tissue regeneration and repair

Transplantation of progenitor cells and gene therapy to induce Müller glia (MG) cells for retinal cell regeneration is a key strategy to treat human retinal disorders (77). Different types of EVs carry various cargo materials for cell-to-cell communication and biological signaling pathways, inducing specific cell regeneration (78). After anesthetizing D. rerio with 4% tricaine, a cornea incision near the pupil was made, and a vitreous injection of C6 glioma cell-derived small EVs (sEVs) (0.5 µL of 10^8^ to 10^11^ sEVs/mL) near the incision site induced MG proliferation with significant ascl1a gene expression (79). The binding of sEVs with MG might induce signal transduction to initiate proliferation, or sEVs might be endocytosed to activate toll-like receptors to initiate retinal regeneration. Beyond retinal regeneration, EV-mediated regenerative processes have also been reported in skeletal tissues and internal organs. These findings suggest that EVs are not limited to neural repair but may function as universal mediators of tissue remodeling and regeneration across diverse biological systems in fish. Osteoclasts (OC) and osteoblasts (OB) are responsible for bone degradation and formation, respectively. Communication disruption between OB and OC causes many metabolic and genetic bone diseases, with OB regulating OC formation and activity through signaling molecules (80). The RANKL-RANK signaling pathway activates TNF receptors to regulate OC formation, survival, and activity, and zebrafish scale fracture stress produces multinucleated OC, while OB-derived EVs contribute to OC activity and differentiation (**Table 1**) (88). Additionally, osteoblastogenesis and osteoclastogenesis largely depend on the release and uptake of EVs, highlighting their role in the cellular mechanisms underlying bone formation. However, the regenerative capacity of EVs is not limited to skeletal tissues. EVs can also facilitate the repair of internal organs damaged by environmental toxicants and physiological stressors. Cadmium (Cd) is toxic to fish and binds with metallothioneins, resulting in cell toxicity after accumulation in localized organs like the liver, kidney, and others. Cd toxicity is severe in the kidney, lowering calcium absorption required for bone formation and maintenance (100). Purified EVs from human bone marrow stem cells injected into Cd toxicity-exposed medaka (*Oryzias latipes*) repaired kidney membranes (apical and basolateral), glomerulus cells, proximal tubule mitochondria, and bone deformation (94). Zebrafish melanoma cell-originated EVs contain CD9 but not CD63, and these EVs have been shown to promote the regeneration and repair of various tissues (86). Tetraspanins like CD63, CD9, and CD81 are considered exosome markers (101). Zebrafish caudal fin regeneration has been reported to involve EV cargo proteins and mRNA (95). Although EVs facilitate cell-to-cell communication, the specific mRNA responsible for fin regeneration has not been confirmed. However, epidermis secreted Shh and Wnt proteins, transported by EVs, contribute to tissue regeneration (102, 103). Although several studies on EV-mediated tissue regeneration have used zebrafish or medaka as experimental models, some administered EVs were derived from non-fish sources, including mammalian cells or cell culture systems. Therefore, this section presents fish model-based evidence for EV-mediated tissue repair rather than direct evidence for aquaculture applications of fish-derived EVs.

**Table 1:** Extracellular vesicle (EV) potentialities analysis in different aquaculture species.

Fish species	Identified EVs	Types/Content of EVs	Effect of EVs on fish	Ref.
Zebrafish (*Danio rerio*)	Small EVs from C6 glioma cells	138 proteins	Induce Muller glia cells to regenerate lost neurons and photoreceptors	(79)
Tongue sole (*Cynoglossus semilaevis*)	Seminal plasma exosomes	Piwi-interacting RNAs	piRNAs regulate sex development	(81)
*D. rerio*	Exosomes	Early endosomes, late endosomes, and multivesicular bodies	EVs distribution in gut cells	(82)
*D. rerio*	Multivesicular bodies and their exosomes	Not mentioned	Exosomes distribution in intestinal immune cells	(83)
Rainbow trout (*Oncorhynchus mykiss*)	Plasma exosomes	MicroRNAs (miR)-21a-3p, let-7a-5p, miR-143-3p, and miR-29a-3p	Air exposure stress increased stress and DNA methylation related miR transcription	(84)
*O. mykiss*	Plasma exosomes	Heat shock protein (Hsp)-70	Heat stress and cortisol increased and decreased Hsp70 transcription, respectively	(85)
*D. rerio*	Tumor EVs	Pro-tumoral and metastatic proteins, mRNAs, miRNAs, and other non-coding RNAs	Melanoma EVs are responsible for metastatic outgrowth	(86)
Salmon (*Salmo salar*)	Head kidney leukocytes exosomes	Major histocompatibility complex class II	Adaptive immune response regulation	(87)
*D. rerio*	Osteoblasts EVs	mCh^+^ Hoe^high^, mCh^+^ Hoe^low^, GFP^low^, and GFP^high^ fraction	Regulation of osteoclasts differentiation via Rankl signaling	(88)
*S. salar*	Serum EVs	180 proteins and 35 are unique in *Piscirickettsia salmonis* infected fish	Infected cells release stimulatory molecules to promote immune response	(89)
Atlantic cod (*Gadus morhua*)	Serum and mucus EVs	C4-like protein	Tissue remodelling and immune defences	(90)
Tuna	*Lactobacillus plantarum* EVs	Not mentioned	Anti-pathogenic activity against *Shewanella putrefaciens* and shelf- life elongation	(91)
*G. morhua*	Mucosal EVs	Peptidylarginine deiminases, C reactive proteins, complement component C3	Regulation of mucosal immunity	(92)
*G. morhua*	Serum EVs	miR, complement component C3, C-reactive protein, and histone H3	Environmental temperature modulates miRNA and protein	(30)
Atlantic halibut (*Hippoglossus hippoglossus*)	Serum EVs	Complement component C3, C4 and CRP, pentraxin protein	Deiminated proteins regulate gene regulation, immune response, metabolic and developmental processes	(93)
Medaka (*Oryzias latipes*)	Human bone marrow mesenchymal stem cells EVs	Not mentioned	Damage repair in kidney cells and improved survival	(94)
*D. rerio*	Fin exosomes	Not mentioned	Cell to cell communication for caudal fin regeneration	(95)
*S. salar*	Head kidney leukocyte EVs	19 differentially enriched miRs including ssa-miR-146a, ssa-miR-155, ssa-miR-731, ssa-miR-125b and others	Macrophage differentiation, maturation and immune response	(96)
*Cynoglossus semilaevis*	Serum exosomes	270 differentially expressed miRs including Aca-miR-155-5p, Bta-miR-132, Mmu-miR-155-5p, Aca-miR-212a-5p, Dre-miR-205-3p, Tgu-miR-155-5p, and oan-miR-147 are expressed in male	7 signature miR index for male and female identification	(36)
*D. rerio*	Brain endothelial cell exosomes	Not mentioned	siRNA inoculation increases permeability in brain endothelial cells	(97)
Grass carp (*Ctenopharyngodon idellus*)	kidney cells exosomes	1297 proteins	Cellular response to Grass carp reovirus infection and survival	(39)
*C. semilaevis*	Seminal plasma exosomes	Among 518 miR 31 as signature candidate, including dre-miR-141-3p, dre-miR-10d-5p, ssa-miR-27b-3p, and ssa-miR-23a-3p	miR act as biomarker of males and pseudo-males.	(98)
*C. semilaevis*	Mucus exosomes	Lower dre-miR-184 and dre-miR-205-5p, and higher dre-miR-100-5p expression in infected fish	miRNAs expression differentiates diseased and healthy fish	(38)
*C. semilaevis*	Skin mucus exosomes	Immunity or infection related 45 piRNAs among them 20 and 25 piRNAs showed high and low expression, respectively in sick fish.	piRNAs are biomarkers of diseased and normal fish	(99)
*C. semilaevis*	Skin mucus exosomes	Total 1531 proteins, and in sick fish among 359, 161 were up-regulated and 198 were down-regulated.	Protein and miR are biomarker of sick and healthy fish	(44)

### 4.2 EVs potentiality in fish immune responses regulation

Fish adaptive immune response depends on the processing of pathogen-derived peptides within the major histocompatibility complex class II (MHCII) on the surface of antigen-presenting cells (APCs). Monocytes, macrophages, and dendritic cells (DCs) express moderate levels of MHCII, and DCs secrete MHCII within exosomes containing costimulatory molecules (104). Salmon (*Salmo salar*) leucocytes produce EVs with exosome markers, and APCs release high concentrations of MHCII. Salmon head kidney leucocyte-derived MHCII were Ig-negative, and APCs originated from MHCII-containing exosomes (87). Moreover, the upregulated transcription patterns of different immune genes, along with lower expression of CpG-B than LPS, indicated the involvement of various genes in the regulatory mechanism of the adaptive immune response. Macrophages play crucial roles as immune response regulators, including the production of reactive molecules, expression of pro- and anti-inflammatory proteins, and phagocytosis (105). Salmon head kidney leucocyte-originated EVs miRNA sequencing identified 19 miRNAs on day 1 compared to day 5, among which ssa-miR-146a, ssa-miR-155, and ssa-miR-731 were responsible for macrophage differentiation, maturation, and immune responses (**Table 1**) (96). Moreover, EVs contain teleost-specific ssa-miR-2188, ssa-miR-731, and ssa-miR-21, which are responsible for macrophage activation in vertebrates.

A well-defined interaction among intestinal epithelial cells, mucus layers, and luminal contents is essential for maintaining immunological homeostasis to ensure protection against pathogenic microbial action (106, 107). Early endosomes, late endosomes, and heterogeneous MVB exosomes are mostly distributed in the apical and basal cytoplasm, super nuclear part, and midgut, respectively, in D. rerio intestinal absorptive cells (**Table 1**) (82). In the same species, Bai et al. (83) first reported the presence of mitochondria-rich cells (MRCs) in intestinal epithelial cells, classified as “open-type” and “close-type” according to their connection to the intestinal lumen. MVBs and their exosomes were distributed at different concentrations in intestinal immune response mediator cells, including MRCs, high endothelial cells, goblet cells, and lymphocytes. The complement system mechanism is considered the first line of immune defense against pathogenic invaders and contributes to the necrotic and apoptotic cell clearance, balancing the inflammatory and homeostatic processes (108, 109). Atlantic cod serum and mucus possess complement components C3, C4, CRP-I, and CRP-II proteins, deiminated C3, and peptidylarginine deiminases (PADs) enzymes involved in immunological, physiological, and pathophysiological activities. Cod serum and mucus EVs contain lower deiminated protein levels than their originating sources, and serum EVs contain higher levels of normal and deiminated C4 proteins than mucus (90). Magnadóttir et al. (92) reported that cod mucosal EVs carry PADs, deiminated C3, and various types of immune and cytoskeletal proteins to maintain mucosal immune responses. Atlantic halibut (*Hippoglossus hippoglossus*) serum EVs cargo contain 124 proteins, 15 of which are confirmed as deiminated proteins (93). Results also confirmed C3 was exported in higher amounts in EVs compared to C4, whereas in EV fraction, protein cargo, gene transcription, immune, and metabolic pathways, deaminated C4 was less detected than C3. Proteomic analysis of grass carp (*Ctenopharyngodon idellus*) reovirus-infected cells identified a total of 1,297 exosome proteins interacting with 10 significant signaling pathways (**Table 1**) (39). Moreover, the GW4869 exosome inhibitor suppressed the VP7 expression of infected cells, indicating the role of EVs in infection response to maintain the life cycle of the infection-causing virus. Further research is needed to analyze the engagement of different EV components in fish immunological functions.

### 4.3 EVs property in fish sex identification

The half-smooth tongue sole (*Cynoglossus semilaevis*) is a significant species in aquaculture, notable for its pronounced sexual dimorphism, with females exhibiting larger body sizes and faster growth rates than males (110). This species also presents a unique phenomenon known as pseudomales, which possess a karyotype similar to females but exhibit physiological characteristics of males. Identifying pseudomales is crucial for managing male-to-female ratios in breeding and aquaculture. Epigenetics and post-transcriptional gene regulation are linked to piwi-interacting RNAs (piRNAs). These piRNAs, ranging from 26–31 nt in size, differ from the smaller mRNAs (21–24 nt) due to their higher complexity, lack of sequence conservation, and increased concentration in gametes (111). In a recent study on *C. semilaevis*, six sex-related signature piRNAs were identified in seminal plasma exosomes from males and pseudomales (81). Bioinformatics analysis identified eight piRNAs commonly shared among 37 sex-related, 15 DNA methylation-related, and 10 transport-related piRNA sets (**Table 1**) (81). The expression pattern of these six sex-related piRNAs was significantly higher in males compared to pseudomales, making them potential male-specific biomarkers for this species. Endogenous miRNAs and other non-coding RNA molecules, typically 22 nt long, are involved in gene transcription regulation related to cell growth, development, differentiation, and apoptosis (112). The transfer of miRNAs via exosomes plays a critical role in cellular communication and post-transcriptional regulation. Extensive investigations into seminal plasma exosomes have identified signature miRNAs (dre-miR-141-3p, dre-miR-10d-5p, ssa-miR-27b-3p, and ssa-miR-23a-3p), with higher expression in males compared to pseudomales (98). Among the 270 serum exosomal miRNAs identified in the early life stages of male and female *C. semilaevis*, 20 were recognized as signature miRNAs. Of these, seven were highly expressed in males compared to females (36). These seven markers, along with 27 genes, were ultimately verified for their role in sex differentiation, providing valuable targets for aquaculture practices aimed at optimizing male and female ratios.

### 4.4 EVs involvement in fish health and disease diagnosis

In recent years, bacterial pathogens have become increasingly common in aquaculture, with *V. harveyi* emerging as one of the most prevalent infectious etiologies in tongue sole. The fish body surface mucus acts as an immune barrier for infection control, and mucus exosomes play a crucial role by containing miRNAs and proteins that act as anti-infectious agents during fish infectious diseases (113, 114). In a study involving nine month-old *C. semilaevis*, the challenge with 100 µL of 1 × 10^6^ CFU/mL *V. harveyi* led to increased transcription levels of mucus exosomal dre-miR-100-5p and decreased dre-miR-184 and dre-miR-205-5p compared to healthy fish, providing insights into fish health status (**Table 1**) (38). Zhao et al. (99) similarly isolated mucus piRNA from *V. harveyi-*infected and normal *C. semilaevis.* The number of clean reads in sick and normal fish was 105,345 and 455,144, respectively. Among the 45 candidate piRNAs, 20 demonstrated higher expression, while 25 showed lower expression in infected fish. Notably, five biomarker piRNAs-piR-mmu-16401212, piR-mmu-26829319, and piR-gga-244092 were lower, while piR-gga-71717 and piR-gga-99034 were higher in infected fish compared to the non-infected group. Skin mucus analysis identified 1,531 credible proteins related to structural, metabolic, and immune functions. In *V. harveyi-*infected fish, among the 369 expressed proteins, 161 were upregulated and 198 were downregulated compared to healthy tongue sole (**Table 1**) (44). Additionally, miRomics of *V. harveyi-*infected fish skin mucus exosomes revealed upregulation of mRNA related to ferritin, the toll-like receptor 5S protein, and calcium transporting ATPase, while Histone H2B and Eukaryotic translation initiation factor 5A showed contrasting expression patterns. In zebrafish, tumor-derived EVs stained with MemBright were injected into early embryos (2 days old). These labeled EVs did not adhere to red blood cells but were taken up by endothelial cells, patrolling macrophages, and immobile Gata1-positive cells (86). Interestingly, macrophage motility decreased after exposure to tumor EVs, which were subsequently stored in degradative compartments. However, tumor EVs, activated macrophages, promoting metastatic outgrowth. Another study focused on healthy and *Piscirickettsia salmonis-*infected salmon serum EVs quantifying 180 proteins. Among these, 35 were unique to infected fish (**Table 1**) (89). Small interfering RNA (siRNA) is known for its high specificity and efficiency with low toxicity. siRNA, typically liposomes, can be delivered into cells through endocytosis and fusion with the cell’s lipid bilayer. Yang et al. (97) inserted siRNA into brain endothelial cell exosomes, significantly enhancing rhodamine 123 permeability. Exosomes facilitated siRNA delivery across the blood-brain barrier, reducing fluorescence in zebrafish (fli1:GFP) xenotransplanted brain tumors after applying exosomes loaded with vascular endothelial growth-related siRNA. Additionally, Lee et al. (91) reported that *Lactobacillus plantarum* EVs possess anti-bacterial potential against pathogenic *Shewanella putrefaciens.* These EVs lowered oxidation reactions, total volatile nitrogen, and peroxidase values, resulting in prolonged tuna shelf life.

### 4.5 EVs properties as fish stress marker

The composition of EVs is influenced by factors such as hypoxia, irradiation, and heat; therefore, EVs may serve as potential stress biomarkers in living animals (115). However, there has been limited research quantifying the expression patterns of miRNAs in circulating EVs in response to environmental stress in aquaculture species. Enriched acetylcholinesterase and heat shock protein (Hsp)-70 are considered EV biomarkers. For instance, rainbow trout exposed to +12 °C (compared to the normal 13 °C) for 1 h showed increased plasma cortisol levels at 4 h during recovery (85). *In vitro* hepatocytes exposed to +15 °C for 1 h exhibited elevated exosomal Hsp-70 transcription over 24 h, while cortisol treatment decreased Hsp-70 expression in both normal and heat-stressed hepatocytes. In another experiment, *O. mykiss* exposed to acute air stress for 3 min was allowed to recover for 1, 3, and 24 h. Plasma cortisol levels increased at 1 and 3 h, returning to control levels by 24 h. However, higher plasma glucose levels remained unchanged across different time points. Stress-related miRNAs (miR-21a-3p, Let-7a-5p, and miR-143-3p) and DNA methylation-related miRNA (miR-29a-3p) showed upregulation in EVs during recovery, while their expression decreased in the liver and head kidney (**Table 1**) (84). In the case of *G. morhua* cultured for 1.5 years at 4 °C and 9 °C, higher temperatures reduced mucus EV concentration, complement component C3, and neutrophil extracellular markers (such as C3, CRP, and histone H3) (30).s Additionally, elevated rearing temperatures led to increased transcription of inflammation-related miRNAs (miRNA-21) and stress-related miRNAs (miRNA-155), while growth-related miRNA-206 was more abundant at lower temperatures.

## 5 *In vivo* OMVs properties in fish

### 5.1 OMVs potentiality in fish mortality protection

*F. noatunensis* is responsible for causing infectious francisellosis in the aquaculture industry, primarily consisting of two subspecies adapted to host temperature: *F. noatunensis* ssp. *orientalis* (FNO) in warm water and *F. noatunensis* ssp. *noatunensis* (FNN) in cold water (116, 117). FNO has been shown to cause aggressive mortality, with about 95% tilapia mortality, and FNN follows a similar mortality pattern in different aquaculture species (118). No commercial vaccine has been produced against this infectious disease, but the application of OMVs from this gram-negative pathogenic bacterium may be a potential solution for francisellosis protection.

Anesthetized *D. rerio* challenged with PBS, PBS + 10^8^ CFU, and PBS + 10^9^ CFU FNN demonstrated fish mortality in a dose-dependent manner (**Table 2**) (37). After 32 days of immunization with 40 µg of FNN OMVs injection and then artificial infection with 10^8^ CFU, immunized fish showed similar survival percentages to the non-infected control group with no adverse effects. Moreover, FNN OMV vaccination decreased the bacterial burden in the spleen after 2 weeks post-infection at 10^6^ genomic equivalents and resulted in a lesser granulomatous response in the kidney. Additionally, *Francisella* virulence factors IgIC, PdpD, and PdpA were identified in the OMVs. Similarly, 20 µg FNO OMVs immunization decreased the mortality of *D. rerio* after 10^6^ CFU FNO immersion challenge, and OMVs-vaccinated fish demonstrated fewer granulomatous structures in the spleen compared to the unvaccinated fish group after pathogenic challenge (**Table 2**) (120). Moreover, virulence factors IglC and Iglb were found in OMVs, and mRNA transcription of cytokine (*tnf-α, il-1β, inf-*γ) and anti-viral (*mhcii, mneg1.1, and ighm*) genes indicated the immunogenic properties of FNO OMVs.

**Table 2:** Outer membrane vesicles (OMVs) properties analysis in different aquaculture species.

OMVs origin	Fish species	Content of OMVs	Effects on fish	Ref.
*Francisella noatunensis* subspecies *noatunensis*	Zebrafish (*Danio rerio*)	IglC, PdpD and PdpA	Protection against francisellosis, reduced bacterial burden and granulomas formation	(37)
*Flavobacterium psychrophilum*	Rainbow Trout (*Oncorhynchus mykiss*)	Among 2046 transcripts 168 were unique, histone like DNA binding protein, cell wall-associated hydrolase gene	Shaping host microbe relationship, virulence and pathogenesis	(119)
*F. noatunensis* subspecies *orientalis*	*D. rerio*	IglC and IglB	Transcription of cytokine *tnf-α*, *il-1β*, and *ifn-γ*, as well as anti-viral *mhcii*, *mpeg1.1*, and *ighm* genes.	(120)
*F. noatunensis* ssp. *orientalis* and *F. noatunensis* ssp. *noatunensis*	Nile Tilapia (*Oreochromis niloticus*) and Atlantic Cod (*Gadus morhua* L.)	Not mentioned	Transcription of *tnf-α*, *il-1β*, no protection against francisellosis and IgM production	(52)
*Photobacterium damselae* subsp. *piscicida* MT1415	Sea bass (*Dicentrarchus labrax*)	TonB-dependent receptor, putative lipase, Ig-like domain-containing protein	Mother cell protection from bactericidal activity and prevent bacterial infection	(121)
*Piscirickettsia salmonis* LF-89	*D. rerio*	452 proteins including porin F, hemolysin, *Bordetella pertussis* toxin subunit 1, and enterotoxin alpha chain	Total protein characterization of OMVs	(122)
*P. salmonis*	Atlantic salmon (*Salmo salar*)	Not mentioned	Transcription of *il-10* and *tgf-β* and IgM level as inflammatory response	(34)
*F. psychrophilum*	*O. mykiss*	sRNAs, soFE013584 and soFE002123	Increment of host immune defense	(55)
*Yersinia ruckeri*	*O. mykiss*	Not mentioned	Formalin killed whole bacteria showed better mortality protection than OMVs	(123)
*P. salmonis*	*D. rerio*	Not mentioned	*mpeg1.1*, *tnf-α*, *il-β*, *il-10*, and *il-6* expression along with IgM production	(124)

In the same species, the 10^8^ CFU *P. salmonis* LF-89 challenge demonstrated lower survival rates in the control group compared to the 20 µg OMVs immunized fish group at 56 days (124). At 7 days post-challenge, non-immunized fish showed higher bacterial concentrations in the intestine and liver and lower IgM secretion than the immunized fish. Moreover, the transcription of different immune genes like *mpeg 1.1, tnf-α, il-1β*, and *il-6* in the kidney and spleen confirmed the potential of the OMV vaccine.

In *O. mykiss*, immersion with formalin-killed *Yersinia ruckeri* (4 × 10^9^ CFU/mL) and this pathogen-derived OMVs (≈ 2 µg) increased the relative percentage of survival (RPS) compared to the control against a 6 × 10^6^) CFU live *Y. ruckeri* challenge (123). Significantly higher lysozyme, % lymphocyte, antibody titer, and RPS were found in the formalin-killed whole cell group relative to the control and OMV. However, Mertes et al. (52) injected FNO and FNN OMVs in *O. niloticus* and *G. morhua*, respectively, to characterize francisellosis protection in these two important commercial species. In tilapia, OMVs immunization with 400 µg increased the transcription levels of *tnf-α* and *il-1β*, but there was no significant production of IgM, kidney bacterial burden reduction, or protection from francisellosis mortality. Similarly, FNN OMVs did not produce anti-FNN IgM after 30 and 61 days post-injection, showed no toxic signs of OMV immunization, and did not induce any immune gene transcription in the various organs of *G. morhua*.

Teixeira and colleagues (121) tested the vaccine potential of OMVs derived from *Photobacterium damselae* subsp. *piscicida* MT1415 in sea bass (*Dicentrarchus labrax*). During *in vitro* characterization, vesicle related proteins were identified, and OMVs demonstrated anti-bactericidal activity against antimicrobial peptides to protect the parent cell. *In vivo* vaccination with OMVs increased the RPS by 35.1% and 38.2% compared to the control (PBS) when challenged against live MT1415.

### 5.2 Fish immune modulation and pathogenic property of OMVs

Piscirickettsiosis is a type of rickettsial septicemia that affects different localized organs, including the gills, liver, kidneys, ovaries, and intestines of *S. salar*, coho salmon (*O. kisutch*), and *O. mykiss* (125, 126). This disease causes significant losses in the salmon industry. The facultative gram-negative *P. salmonis* is the primary cause of piscirickettsiosis, making it one of the most important diseases in Chile’s mariculture industry (127). *P. salmonis* strain LF-89 produces OMV containing 452 cytoplasmic proteins that aid in bacterial survival. These proteins include hemolysin-related virulence proteins, *Bordetellapertussis* toxin subunit 1, and the heat-liable enterotoxin alpha chain (**Table 2**) (122). Artificial infection experiments using OMVs (at 40 µg) demonstrated 5% and 15% higher mortality in *D. rerio* compared to 10 µg and 20 µg OMVs, respectively. This highlights the role of OMVs in fish pathogenesis, with the highest mortality observed in the group challenged with 10^9^ CFU of live *P. salmonis* LF-89 cells.

In worldwide salmonid aquaculture, the gram-negative bacterium *F. psychrophilum* is responsible for causing bacterial cold water disease (BCWD) (128). The pathogenesis of *F. psychrophilum* involves biofilm production, survival in harsh environments, multiple transmission routes, and the lack of an effective vaccine against this bacterium (129). *F. psychrophilum* OMVs contain cell wall-associated hydrolase enzymes that facilitate cell autolysis and transfer inner cargo into host cells during interactions. Comparative transcriptomic analysis revealed that *F. psychrophilum* whole cells and OMVs share1878 transcripts, while 168 transcripts are unique to OMVs. When BCWD-resistant and susceptible *O. mykiss* were infected with F. psychrophilum OMVs, several genes were downregulated in the resistant fish line. These genes include Fe-S cluster assembly ATPase, ferredoxin, and ferritin-like domain-containing proteins, confirming the host-pathogen relationship, pathogenesis, and virulence (**Table 2**) (119). *In vitro* experiments using the trout cell line RTgill-W1 treated with *F. psychrophilum* OMVs demonstrated cell autolysis and differential gene expression after 24 h. Furthermore, OMVs from *F. psychrophilum* infection in both resistant and susceptible rainbow trout revealed 116 bacterial sRNAs. Among these, sRNAs soFE013584 and soFE002123 were associated with telomere protection. An upregulation of the anaphase-promoting complex subunit 13 gene and reciprocal expression of 21 OMVs sRNAs indicated the involvement of phagocytic, endocytic, and antigen presentation pathways in fish immune responses boosted by OMVs (55). In *S. salar*, immunization with 40 and 400 µg of *P. salmonis* OMVs induced immune-modulating IgM production. Additionally, transcriptional changes in interleukin-10 (*il-10*) and transforming growth factor beta (*tgf-β*) were observed in the head kidney, liver, and spleen after immunization with 10 and 30 µg of *P. salmonis* OMVs (34).

## 6 *In vitro* fish pathogenic bacterial OMVs potential

### 6.1 OMVs action in cell cytotoxicity

*P. salmonis* typically infects and multiplies within macrophages, utilizing clathrin for uptake into SHK-1 cells by disrupting the actin cytoskeleton (130, 131). This infectious pathogen secretes OMVs ranging from 25 to 145 nm, which are structurally identical to those of other fish pathogens like FN and *V. anguillarum* (132, 133). The outer membrane protein of *P. salmonis* is identical to the membrane protein found in OMVs. Western blot and LC-MS/MS analysis confirmed the presence of bacterial chaperon proteins in these vesicles. Furthermore, changes in CHSE-214 cell elongation and detachment after treatment with *P. salmonis* OMVs differed from live pathogen application. Cell vacuolization observed in CHSE-214 cells indicates the active virulence and cytopathic properties of OMVs (**Table 3**) (134). The average size of OMVs from three pathogenic strains of *V. ordalii* (Vo-LM-18, ACTC 33509, and *V. anguillarum*, ACTC 43307) is approximately 0.215 ± 0.6 µm, with no significant similarity in total and external proteins. A comparison between two *V. ordalii* strains revealed the highest degree of similarity in total protein between 37 and 50 kDa. The hemolytic activity of OMVs is responsible for *V. ordalii* infection in salmon culture (53). Tenacibaculosis, a common infectious disease, is caused by *Tenacibaculum dicentrarchi* in species such as *O. mykiss*, *O. kisutch*, and red conger eel (*Genypterus chilensis*) (139). The average size of OMVs from these three *T. dicentrarchi* strains (TdCh05, QCR29, and CECT 7612) ranges from 82.25 to 396.88 nm. These OMVs share a common pattern of five dissimilar proteins. Purified OMVs demonstrated cytotoxic effects on *O. mykiss* head kidney macrophage cells due to their pathogenic nature, inherited from the parental bacteria (42).

**Table 3:** Different fish pathogenic bacteria originated OMVs property analysis *in vitro*.

OMVs origin	Size/Content of OMVs	Identified property	Ref.
*Piscirickettsia salmonis* LF-89	Size 25–145 nm, bacterial chaperonin Hsp60 protein	Cytopathic effect on CHSE-214 cell	(134)
*Aeromonas hydrophila* and *A*. *salmonicida*	Size 20–300 nm	Presence in intestinal apical surface of epitheliocytes	(135)
*A. veronii* biotype *sobria* 104, 106, and 102 strain	Not mentioned	Biofilm formation	(43)
*Yersinia ruckeri*	Not mentioned	Pathogenic antigen OmpA and OmpF can be expressed in *E. coli* vector	(136)
*Renibacterium salmoninarum*	P57/Msa and P22 protein, New Lipoprotein C/Protein	Involvement in host-pathogen interaction and OMVs formation	(137)
*Vibrio ordalii* Vo-LM-18 and ATCC 33509	Not mentioned	Size 0.19 to 1.8 µm, protein similarity between 37 and 50 kDa, and haemolytic enzyme activity	(53)
*Tenacibaculum dicentrarchi* TdCh05, QCR29, and CECT 7612	Not mentioned	Size 82.25 to 396.88 nm, protein patterns are not identical, and cytotoxic effects on *O. mykiss* head kidneys cell	(42)
*T. maritimum* SP9.1	PorP, PorT, SprA TonB-dependent transporters, and T9SS proteins	Biofilm formation, cytotoxic effect, and ulcerative and hemorrhagic effect on Senegalese sole	(138)

### 6.2 OMVs involvement in biofilm production and vesicle regeneration

Pathogenic bacterial biofilms play a crucial role in protecting against antimicrobial agents, adverse conditions, and various destructive processes. Toyofuku et al. (140) highlighted that extracellular polymeric substances, including polysaccharides, extracellular DNA molecules, nucleic acids, and certain matrix proteins, are closely associated with biofilm formation in pathogens. Among the pathogenic strains of *A. veronii* biotype *sobr*02, 104, and 106), only strains 104 and 106 exhibited improved biofilm formation compared to 102 after incorporating their respective OMVs. It is assumed that strains 104 and 106 possess receptors that facilitate OMV binding, leading to signal transduction for biofilm formation (**Table 3**) (43). The OMVs of the pathogenic bacterium *Tenacibaculum maritimum* contain TonB-dependent siderophore transporter T9SS proteins, such as ProP, ProT, and SprA, along with soluble extracellular products. These OMVs commonly share 166 proteins. Additionally, the addition of OMVs to *T. maritimum* increased biofilm production relative to the pathogen alone. Notably, OMVs also exhibited cytotoxic effects on epithelioma papulosum cyprinid cells and caused ulcerative hemorrhage in senegalase sole (*Solea senegalensis*) (138). Salmonid bacterial kidney disease, caused by *Renibacterium salmoninarum*, and Kroniger et al. (137) reported P57/Msa and P22 proteins within bacterial OMVs. A proteomic analysis revealed a higher P22 concentration in OMVs, and these vesicles contain four cell wall hydrolase enzymes, including a novel lipoprotein C/protein (60 kDa) family, contributing to the pathogenesis of this bacterial OMV formation.

### 6.3 OMVs other properties

Yersiniosis, associated with *Y. ruckeri*, leads to enteric red mouth disease, primarily affecting farmed and wild salmon. Vaccines against bacterial pathogens typically consist of dead whole cells, formalin-inactivated bacteria, or live-attenuated or subunit vaccines (141, 142). However, OMV applications pose challenges due to their reported involvement in bacterial pathogenesis without specific antibody production or immunomodulation in fish (143, 144). To address this, a recombinant OMV approach involves inserting *Y. ruckeri* outer membrane proteins (OmpA and OmpF) into *E. coli* (**Table 3**) (136). Initially, *E. coli* genes (OmpA, OmpC, OmpF, and lamB) were knocked down, and *Y. ruckeri* antigen was inserted, with expression confirmed via SDS-PAGE. Lusta and Kozlovskii (135) characterized OMVs from *Aeromonas hydrophila* and *A. salmonicida*, ranging in size from 10 to 300 nm. *In vitro* studies confirmed the presence of OMVs in the glycocalyx between the intestinal microvilli and enterocytes.

## 7 Research gap, future prospects, and concluding remarks

Over the past five years, EVs have played a crucial role in human drug delivery, and their potential application extends to other vertebrates (145). In fish, EVs facilitate cell-to-cell communication, and the transfer of cargo from host to recipient cells modulates different physiological and immunological functions. However, the mechanisms of EV secretion, isolation, and identification from different aquatic animal tissues remain largely unexplored. EV biomarkers not only reflect the physiological and immunological status of aquatic animals but also contribute to processes like wound healing, sex development, bone formation, gene transcription, and macrophage differentiation. While research on engineering EVs for human drug delivery is ongoing, their application in aquaculture species remains understudied, particularly for protecting fish against infectious pathogen invasion.

OMVs have been studied extensively since their discovery in 1960. These non-replicative structures possess immunogenic properties and a smaller size, making them attractive candidates for vaccine development (146). However, a significant challenge in fish vaccine development using OMVs lies in their inherent virulence factors and potential pathogenic effects on the host. OMVs efficacy in modulating fish adaptive immune responses, and acellular vaccines based on OMVs are currently under extensive investigation (54). Cutting-edge research now focuses on biotechnological approaches to engineer pathogenic strains capable of producing OMVs containing multiple antigen proteins for fish immunomodulation. Additionally, inserting outer membrane proteins from OMVs into *E. coli* vectors represents a novel approach for future vaccine development. Nevertheless, several practical challenges remain before EV- and OMV-based technologies can be translated into aquaculture applications. Scalable production systems, standardized quality-control procedures, long-term storage stability, and cost-effective manufacturing processes must be established to ensure their feasibility for disease diagnosis, health monitoring, and vaccine development in aquaculture. Furthermore, future research should focus on developing standardized protocols for vesicle isolation, characterization, and cargo profiling across aquaculture species to improve reproducibility and comparability among studies. From a regulatory perspective, biosafety assessments, environmental risk evaluations, and harmonized approval frameworks will also be required to support the safe and sustainable implementation of EV- and OMV-based technologies in aquaculture.

In this review, we discuss the current landscape of EVs and OMVs, their potential applications in various commercial fish species, and the molecular mechanisms underlying their interactions with hosts. We explore the content of these vesicles, including proteins, mRNA, and pathogenic factors. This report aims to inform disease diagnosis, immune modulation, stress reduction, and vaccine development in aquaculture species. By shedding light on the underexplored field of EVs and OMVs in aquaculture, we hope to inspire intensive research by upcoming researchers.
